# Uncovering a photoprotective polysaccharide from *Gentiana dahurica* via systematic fractionation

**DOI:** 10.1016/j.pscia.2026.100120

**Published:** 2026-04-15

**Authors:** Jiahui Xia, Muhammad Aamir Zohaib, Xiaopeng Su, Wen Ji, Tengfei Hu, Jianqin Zhou, Sheng Tian, Duxin Li

**Affiliations:** College of Pharmaceutical Science, Soochow University, Suzhou 215123, PR China

**Keywords:** *Gentiana dahurica* polysaccharides, Extraction methods, Structural characterization, Antioxidant activity, Photoprotection

## Abstract

*Gentiana dahurica* Fisch. (*G. dahurica*) is a plant native to high-altitude plateaus that has developed unique biochemical adaptations to intense UV radiation. This study aimed to discover novel polysaccharides from *G*. *dahurica* (GDP) with prominent activity against UV-induced photoaging. Three methods were used to extract GDP: hot water (GDP-W), alkaline (GDP-A), and cellulase-assisted methods (GDP-E), with yields of 2.30%, 0.10%, and 0.46%, respectively. GDP-W and GDP-E mainly contained arabinose and galacturonic acid, while GDP-A was mainly composed of arabinose and mannose. Among the three polysaccharides, GDP-A contained a higher proportion of high-molecular-weight fractions. In vitro assays revealed that all GDPs exhibited concentration-dependent antioxidant activity, effectively scavenging DPPH, ABTS, and hydroxyl radicals. GDP-A exhibited the strongest effects. In UVB-irradiated HaCaT cells, all GDPs effectively alleviated the reduction in cell viability and proliferation. They increased the levels of antioxidant enzymes, reduced the levels of inflammatory factors, decreased intracellular ROS accumulation, and inhibited apoptosis. In the zebrafish photoaging model, GDPs significantly reduced the UVA-induced increase in ROS levels. GDP-A exhibited greater efficacy in both antioxidation and inhibiting apoptosis. Furthermore, in a mouse model of skin photoaging, GDP-A ameliorated UVB-induced skin damage. It increased antioxidant enzyme levels, decreased pro-inflammatory cytokines, and suppressed matrix metalloproteinase-3 expression in the epidermis. These changes resulted in higher collagen content and improved therapeutic outcomes. The results indicated the potential use of GDP-A in pharmaceutical and cosmetic applications and provided new insights into the impact of isolation methods on discovering bioactive polysaccharides.

## Graphical abstract

**Figure:**
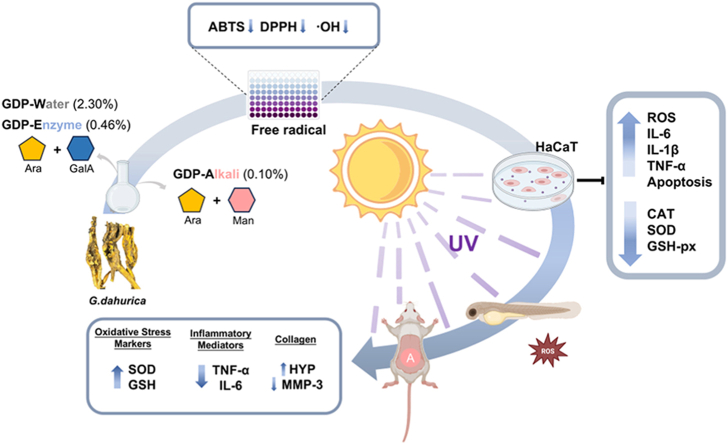

**Table:** Abbreviation list:

UV	ultraviolet
ROS	reactive oxygen species
Man	mannose
Ara	arabinose
GalA	galacturonic acid
DCFH-DA	2′,7′-dichlorodihydrofluorescein diacetate
HYP	hydroxyproline
SOD	superoxide dismutase
GSH	glutathione
IL-6	interleukin 6
TNF-α	tumor necrosis factor-α
Mw	molecular weight
SEM	scanning electron microscopy
HPMC	hydroxypropyl methyl cellulose

## 1 Introduction

Solar ultraviolet (UV) radiation is composed of roughly 90–99% UVA and 1–10% UVB [1]. UV radiation triggers the rapid generation of reactive oxygen species (ROS) upon skin penetration. This process induces DNA damage, collagen breakdown, cell apoptosis, and compromised skin barrier function, which ultimately speeds up photoaging and elevates the risk of skin cancer [2,3]. Individuals living in high-altitude regions are particularly susceptible to intense UV exposure, thus driving the demand for efficient photoprotective sunscreens to alleviate this harm. Traditional chemical sunscreens often bring about side effects like inflammation and allergic responses. Plant-derived photoprotective compounds have attracted growing attention from researchers and industry in recent years.

Polysaccharides, well recognized for their potent antioxidant activity [4], have been confirmed to have protective effects against skin damage caused by UV radiation [5,6]. They have shown great potential for application in skincare products [7]. Importantly, plants growing in high-altitude areas under long-term strong UV radiation may develop unique polysaccharides with protective functions as part of their adaptive mechanisms [4]. Thus, exploring polysaccharides extracted from high-altitude plants for use as photoprotectors holds significance for skincare development and the utilization of local plant resources.

The method of plant polysaccharides isolation is crucial to the discovery of novel polysaccharides. The biological activities of polysaccharides directly depend on their structural features, such as molecular weight, monosaccharide composition, and glycosidic linkages, which are significantly influenced by the isolation methods employed [8,9]. These methods also affect the final yield. Kumar et al. isolated water-soluble polysaccharides using hot water extraction [10]. The obtained polysaccharides demonstrated good flowability and compressibility. Alencar et al. obtained three distinct polysaccharides from winemaking by-products using a series of solvent conditions (neutral, acidic, and alkaline). Among these, alkaline polysaccharide exhibited significant anti-inflammatory activity, which is attributed to its high xylose content [11]. Chen et al. extracted pectin polysaccharides from the peel of pitaya fruits by combining ultra-high pressure and enzymatic treatment. This method improved extraction efficiency and produced polysaccharides with lower molecular weight and esterification degree, while also improving functional properties, including water solubility, thermal stability, and antioxidant activity [12]. Moreover, Zhang et al. used Microwave-assisted enzymatic extraction obtained notably high yield (60.43%) of polysaccharides from *Zizania latifolia* [13]. Li et al. used sequential solvents extraction and obtained five types of cell wall polysaccharides from tomato fruits, which differed in monosaccharide composition and showed variations antioxidant and lipid-lowering activities in vitro [14].

The dried root of *Gentiana dahurica* (*G. dahurica*) is a legitimate source of the traditional Chinese medicine known as Radix Gentianae Macrophyllae. In previous work, our research group isolated two novel polysaccharide fractions (GDP-1 and GDP-2) from the roots of this plant using hot water extraction [15]. The GDP showed promising photoprotective effect in vitro. The present study aimed to develop systematic fractional method to isolated distinct polysaccharides from *G. dahurica*, screen the most promising component in vitro, and validate the efficacy of alleviating UV photoaging in vivo. The developed method provides an insight into the impact of isolation methods on discovering bioactive polysaccharides. This study offers a theoretical basis for the development of *G. dahurica* polysaccharides in pharmaceutical and cosmetic products.

## 2 Materials and methods

### 2.1 Materials and chemicals

The roots of *G*. *dahurica* were sourced from a local apothecary in Xining, China. Cellulase (10,000 U/g) was acquired from Macklin Co., Ltd. (Shanghai, China). The monosaccharide standards, rhamnose (Rha), arabinose (Ara), galactose (Gal), glucose (Glc), mannose (Man), galacturonic acid (GalA), and glucuronic acid (GlcA), and standard molecular weight dextrans were supplied by Aladdin Co., Ltd. (Shanghai, China) and Sigma-Aldrich Co., Ltd. (St. Louis, USA), respectively. Trifluoroacetic acid (TFA) was obtained from Damao Co., Ltd. (Tianjin, China). Several commercial assay kits were utilized: the BCA protein assay kit, DCFH-DA assay kit, and CCK-8 cell counting kit from Beyotime Biotechnology Co., Ltd. (Shanghai, China); HYP, SOD, and GSH assay kits from Solarbio Co., Ltd. (Beijing, China); IL-6 and TNF-α assay kits from Thermo Fisher Scientific Inc. (Massachusetts, USA); and the Annexin-V-FITC/PI staining kit from Vazyme Co., Ltd. (Nanjing, China). All other chemicals and reagents were of analytical grade or higher.

### 2.2 Extraction and purification of *G. dahurica* polysaccharides (GDPs)

#### 2.2.1 Hot water extraction

The method was performed according to Ji et al. [15]. The dry roots of *G. dahurica* were meshed and defatted with 95% ethanol, followed by two rounds of boiling water extraction under reflux for 2 h each. The combined extracts were concentrated via ultrafiltration and then blended with four volumes of ethanol. The mixture was stored overnight at 4 °C. The precipitate was obtained through centrifugation, yielding the crude water-extracted polysaccharide (GDP-W). The residue was dried for later use.

#### 2.2.2 Alkaline extraction

The method referred to Yao et al. [16] and Wang et al. [17]. Half of the dried water-extracted residue was treated with 0.3 M sodium hydroxide containing 0.02 M sodium borohydride at 4 °C for 5 h, repeated twice. After centrifugation and filtration, the supernatant was neutralized with glacial acetic acid. The resulting extract was then concentrated, ethanol-precipitated, and centrifuged. The precipitate was collected as crude alkaline-extracted polysaccharide (GDP-A).

#### 2.2.3 Cellulase-assisted extraction

The method was performed according to Zhang et al. [13]. The remaining half of the dried water-extracted residue was dissolved with buffer (pH 5.0), and 1.5% cellulase (10,000 U/g) was then added. Extracted for 4 h at 50 °C, repeated twice. After centrifugation and filtration, the supernatant was concentrated by ultrafiltration. The concentrate was heated to inactivate enzymes prior to ethanol precipitation and centrifugation. The precipitate was collected as crude enzyme-extracted polysaccharide (GDP-E).

After redissolving in water, all crude polysaccharide fractions (GDP-W, GDP-A, and GDP-E) were decolorized using D301 resin. Proteins were removed using the Sevag method [18]. The resulting solution was dialyzed (3.0 kDa cutoff) against deionized water and lyophilized to yield the final polysaccharides (**GDPs**). The extraction yield (%) was calculated using the following formula:

Extraction yield (%) = (W_1_ / W_0_) × 100%

Where W_1_ and W_0_ are respectively the weight (g) of the polysaccharides obtained and *G. dahurica* powder.

**Fig. 1:**
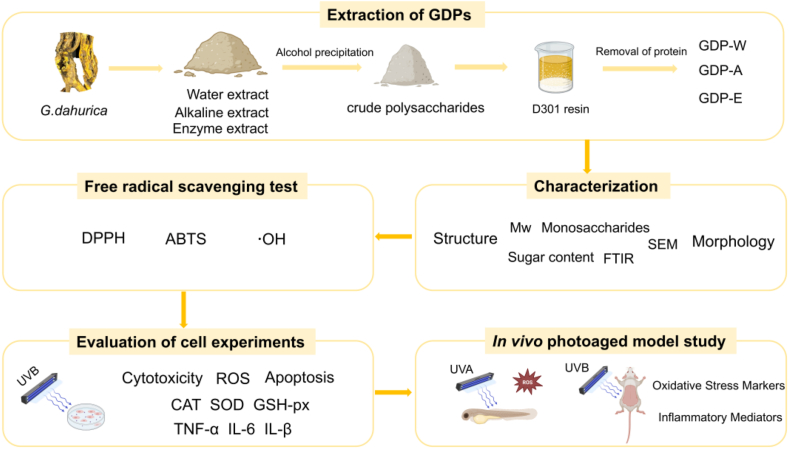
Schematic diagram of the present study.

A schematic representation of the present study is shown in Fig. 1.

### 2.3 Chemical and physical analysis of GDPs

The total sugar content of the GDPs was determined by the phenol-sulfuric acid method using glucose as a standard [19]. The protein content was measured by the Bradford method with bovine serum albumin (BSA) as a reference.

The molecular weights (Mw) of the GDPs were determined by size exclusion chromatography (SEC) on a TSKgel GMPWXL column (300 × 7.8 mm I.D., 13 μm; Tosoh, Japan). The system was equilibrated with a mobile phase consisting of 20% methanol and 80% water containing 50 mmol/L CH_3_COONH_4_. Separation was performed at a flow rate of 0.5 mL/min and a column temperature of 25 °C. Samples (20 μL of 10 mg/mL) were taken for analysis.

Monosaccharide composition was analyzed using an ion chromatograph (IC Professional 850, Metrohm) equipped with pulsed amperometric detection (PAD) and a Carbopac PA1 column (250 × 4.6 mm, 5 μm; Thermo Fisher). The mobile phase consisted of 15 mM NaOH and 150 mM NaOAc, delivered at a flow rate of 1 mL/min. The injection volume was 20 μL. Sample pretreatment followed this protocol: 3 mg of GDPs was dissolved in 0.6 mL water, then added an equal volume of 2 M TFA, and the mixture was hydrolyzed at 100 °C for 12 h. Subsequently, 1 M NaOH was used to neutralize the pH, and the solution was adjusted to a final volume of 5 mL with water. Monosaccharides were identified and quantified using neutral sugar standards within the concentration range of 2.5–100 μg/mL.

The ultraviolet (UV) absorption spectrum of the GDPs solution (0.5 mg/mL) was scanned from 190 to 300 nm using a Shimadzu UV-2600 spectrophotometer [20]. Fourier transform infrared (FT-IR) spectra were obtained on an FT-IR 650 spectrometer (Tianjin, China) employing the KBr pellet method, acquired across the wavenumber range of 400 to 4000 cm^−1^.

Scanning electron microscopy (SEM) imaging of GDPs was carried out using an EVO 18 instrument (ZEISS Group, Oberkochen, Germany). Under vacuum conditions, GDPs samples were evenly spread on conductive adhesive tape, followed by sputtering a gold layer onto the surface. SEM images were captured at multiple magnification levels.

### 2.4 *Invitro* biological activity evaluation

#### 2.4.1 Antioxidant activities of GDPs

The antioxidant activities of GDPs against ABTS, DPPH and hydroxyl radicals were evaluated based on previously established methodologies [21,22].

ABTS radical scavenging assay: a working solution was prepared by reacting 7.0 mM ABTS and 2.45 mM K_2_S_2_O_8_ solutions in equal volumes, followed by 16 h of incubation in the dark. The solution was then diluted so that its absorbance at 734 nm was approximately 0.70. Then, different concentrations of polysaccharide solutions (50 μL) were reacted with the working solution (200 μL) in the dark at 25 °C for 10 min. The absorbance at 734 nm was then measured.

DPPH radical scavenging assay: The sample solution (50 μL) was mixed with an equal volume of a DPPH-ethanol solution. The mixture was kept in the dark at 37 °C for 30 min, and the absorbance was then measured at 517 nm.

Hydroxyl radical scavenging assay: The reaction mixture containing 50 μL of the sample, 50 μL of 9 mM FeSO_4_, and 50 μL of 6.5 mM H_2_O_2_ was allowed to react for 10 min. Then, 50 μL of 9 mM salicylic acid-ethanol solution was added, and the reaction continued for another 30 min. The absorbance was measured at 510 nm.

#### 2.4.2 Cell culture and proliferation assay

HaCaT cells were cultured in basic DMEM medium supplemented with 10% fetal bovine serum and 1% penicillin-streptomycin, and maintained at 37 °C in a 5% CO_2_ atmosphere. The cells were seeded at a density of 1 × 10^5^ cells/mL and allowed to adhere and grow for 24 h. After that, the cells were exposed to GDPs at final concentrations ranging from 10 to 5000 μg/mL (10, 50, 100, 500, 1000, and 5000 μg/mL) and further incubated for 24 h. Thereafter, CCK-8 reagent was added, and the plates were incubated for 1.5 h. The absorbance of each well was measured at 450 nm.

#### 2.4.3 Effect of GDPs on HaCaT cells

HaCaT cells were seeded in 96-well plates at a density of 5 × 10^5^ cells per well and allowed to adhere for 12 h. Then, the cells were treated with GDP-W, GDP-A, and GDP-E at final concentrations of 25, 50, 100, and 200 μg/mL for another 12 h. Prior to UVB irradiation, added PBS solution to cover the bottom. The cells were then exposed to UVB light using a 9 W lamp (peak wavelength: 313 nm) for 12 min, resulting in a total radiation dose of 388 mJ/cm^2^. After that, the cells were washed twice with PBS and then cultured in medium for 12 h. The cell viability was determined by the CCK-8 method.

#### 2.4.4 Analysis of intracellular ROS in HaCaT cells

The levels of ROS in cells after UVB radiation was determined [23]. Briefly, HaCaT cells were seeded in 6-well plates and pretreated with 200 μg/mL of GDPs for a specified period prior to being subjected to UVB irradiation for 12 min. After irradiation, the cells were washed and incubated with 10 μM DCFH-DA for 30 min. After that, images were captured using an inverted fluorescence microscope (TS2R-FL, Nikon, Japan) with excitation at 488 nm and emission at 525 nm, and analyzed using ImageJ software.

#### 2.4.5 Measurement of oxidative stress markers and inflammatory factors

The cell pre-culture procedure was consistent with that described in Section 2.4.3, with GDPs concentrations of 10, 50, and 100 μg/mL. After cell lysis and centrifugation, the protein content was determined using a BCA assay kit. According to the manufacturer's instructions, the levels of CAT, GSH-px, SOD, TNF-α, IL-6, and IL-1β were measured.

#### 2.4.6 Analysis of apoptosis

Cell apoptosis was analyzed using Annexin V-FITC and PI double staining. After treatment with 200 μg/mL GDPs and UVB irradiation, cells were collected and resuspended in 100 μL of staining buffer. According to the manufacturer's instructions, 5 μL of Annexin V-FITC and 5 μL of PI staining solutions were sequentially added and gently mixed. The mixture was incubated for 10 min in the dark, followed by the addition of 400 μL of binding buffer. Apoptosis was assessed using a BD ARAIII flow cytometer (BD Biosciences, CA, USA), and data were processed with FlowJo 10.8.1.

### 2.5 *Invivo* evaluation the effect of GDPs against photoaging

The experimental protocol involving animals received review and approval from the Animal Ethics Committee of Soochow University (approval number: 202401A0119), adhering to the National Research Council's Guide for the Care and Use of Laboratory Animals.

#### 2.5.1 Effects of GDPs on zebrafish embryos

The safety of GDPs on zebrafish embryos was investigated by referring to the method of Cordeiro et al. [24]. The 7 hpf zebrafish embryos were placed in 24-well plates containing 200 μg/ mL GDPs and cultured for 72 h. During this period, recorded the developmental status of embryos every day. Following a 72-h incubation, larval heart rate and body length were measured under an inverted microscope to evaluate GDPs' effects on cardiac function and growth. These outcomes were benchmarked against a control group cultured in blank medium, with data collected from three independent trials (n = 20 per group).

#### 2.5.2 Effects of GDPs on zebrafish embryos exposed to UVA

The measurement of ROS levels was based on a reported procedure [25]. Healthy zebrafish embryos at 7 hpf were randomly allocated into five experimental groups: control, UVA, and three GDP-treated groups (GDP-W, GDP-A, GDP-E). Each group, consisting of 15 embryos, was placed into a 6-well plate. From this stage until 72 hpf, all groups except the control were subjected to daily UVA irradiation (1440 mJ/cm^2^) following pretreatment with either blank medium or 200 μg/mL GDPs. To assess ROS levels, the embryos were subsequently incubated with 20 μg/mL DCFH-DA in the dark for 1 h. Subsequently, the zebrafish were gently rinsed and anesthetized using 0.16% tricaine prior to imaging under a microscope.

#### 2.5.3 Preparation of gels

Initially, 130 mg of hydroxypropyl methyl cellulose (HPMC) was dispersed into deionized water with stirring until complete swelling was achieved. GDP-A (200 mg), ethylparaben (5 mg), and glycerol (500 mg) were added to deionized water and mixed thoroughly to form a homogeneous GDP-A solution. This solution was then gradually incorporated into the HPMC solution. Deionized water was added to adjust the total weight to 5 g, yielding a 4% GDP-A gel. The blank gel was prepared by replacing GDP-A with an equal volume of deionized water.

#### 2.5.4 Establishment of the mouse model of UVB-induced photoaging

The mice were purchased from GemPharmatech Co., Ltd. (certificate number: A202401180152). A total of twenty-four male Balb/c mice were randomly divided into four experimental cohorts (n=6): Control, UVB model, UVB + NC (treated with blank gel), and UVB + GDP-A groups. Prior to the experiment, the dorsal hair of all mice was removed using a depilatory cream. The mice were placed 40 cm below a UVB lamp (peak wavelength 313 nm) and irradiated for 1 h per day over six consecutive days, with a total radiation dose of 2.9 J/cm^2^. Within 30 min after each irradiation session, 80 mg of gel was topically applied to the dorsal skin. Skin recovery and changes were recorded by photography on days 1, 3, 5, and 6 post-irradiations.

#### 2.5.5 Histological section analysis

Fresh tissue samples were dissected and immediately immersed in fixative for at least 24 h. The tissues were then trimmed in a fume hood and dehydrated using a graded ethanol series in an automated tissue processor. The dehydrated tissues were then embedded, sectioned, and stained with hematoxylin and eosin. The stained sections were dehydrated, cleared, and mounted. Finally, the sections were examined and imaged under a microscope.

#### 2.5.6 Biomarker assays in skin tissue

On the terminal day, the skin tissue from the depilated dorsal region of the mice were collected. A portion of skin tissue was homogenized in normal saline at a 1:9 (*w*/*v*) ratio. The homogenate was centrifuged, and the supernatant was collected for analysis. The levels of SOD, GSH, TNF-α, IL-6, hydroxyproline (HYP), and matrix metalloproteinase-3 (MMP-3) were measured using commercial assay kits.

### 2.6 Statistical analysis

Experimental data were processed using GraphPad Prism 9.5, and each experiment was repeated no less than three times. Data were statistically analyzed using one-way ANOVA, and *t*-tests were conducted between groups to determine significance.

## 3 Results and discussion

### 3.1 Extraction of GDPs

**Fig. 2:**
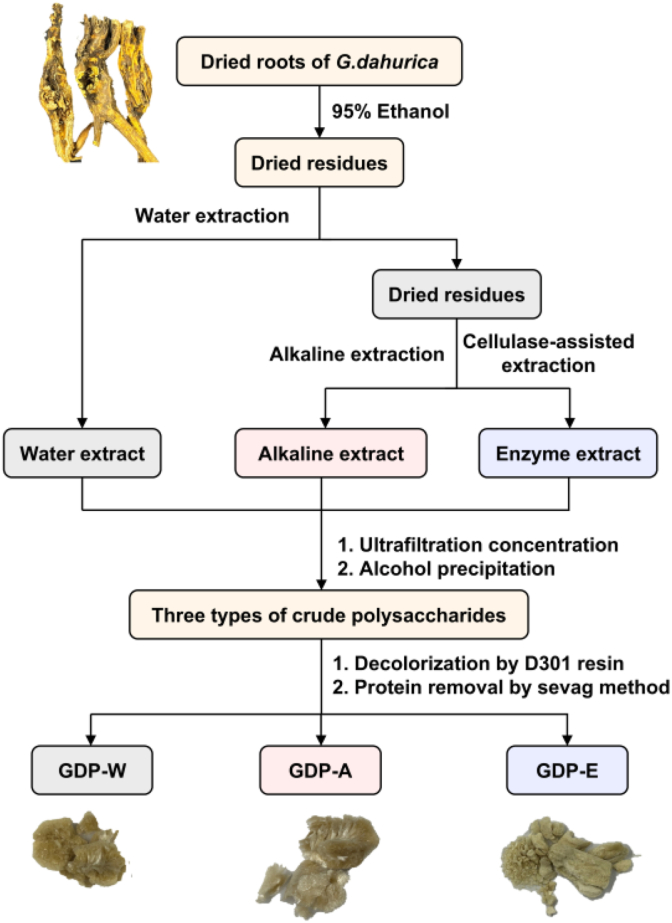
Schematic diagram of the GDP extraction procedure.

The isolation process for the GDPs is illustrated in Fig. 2. The yields of polysaccharides from *G. dahurica* using hot water, alkaline, and cellulase-assisted extractions were 2.30%, 0.10%, and 0.46%, respectively (Table 1). In contrast to previous reports, hot water extraction yielded significantly higher yields than both alkaline and enzymatic methods. This difference may be attributed to the fact that the materials subjected to alkaline and cellulase-assisted extraction were the filter residues remaining after hot water extraction. Under the same conditions, the yield obtained using the enzymatic method was 4.6 times that obtained using the alkaline method. Both enzymatic and alkaline methods can extract polysaccharides that are not accessible via hot water extraction. The notably higher extraction efficiency achieved with the enzymatic method aligns with previous reports, indicating that cellulase effectively breaks down cellulose in plant cell walls and disrupts cellular structures, thereby facilitating the release of polysaccharides [26]. These results demonstrated that the extraction method significantly affects the polysaccharide yield, highlighting the importance of systematically characterizing the composition of GDPs and evaluating their biological activities.

**Table 1:** Yield and composition analysis of GDPs.

Fractions		GDP-W	GDP-A	GDP-E
Yield(%)		2.30	0.10	0.46
Content of total sugar (%)		82.90	81.39	59.70
Content of protein (%)		3.45	4.77	2.92
Monosaccharides composition (%)	Rha	4.36	7.83	5.93
	Ara	41.40	23.19	51.64
	Gal	6.04	8.87	6.87
	Glc	4.14	12.17	3.99
	Man	0.92	44.48	0.99
	GalA	42.18	1.51	29.72
	GlcA	0.95	1.95	0.87

### 3.2 Characterization of GDPs

**Fig. 3:**
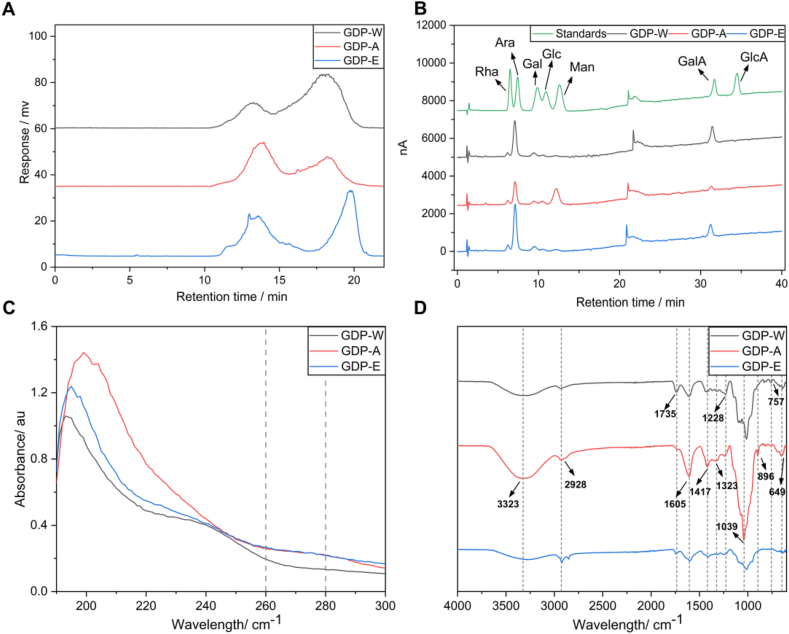
Structural characterization of GDPs. A: Molecular weight; B: Monosaccharide composition; C: UV spectra; D: FT-IR spectra.

The total sugar contents of GDP-W, GDP-A, and GDP-E were determined by phenol-sulfuric acid method (*y* = 2.3527*x* + 0.0740, *R*^2^ = 0.9987), and the results were 82.90%, 81.39%, and 59.70%, respectively. The protein contents, determined using the Bradford method (*y* = 0.7912*x* + 0.0972, *R*^2^ = 0.9980), were 4.48%, 5.73%, and 2.13%, respectively. The UV-Vis spectra of the GDPs (Fig. 3C) showed no obvious absorption peaks in the 260–280 nm range, indicating very low levels of protein and nucleic acid contamination in the polysaccharides obtained by all three methods.

The chromatograms exhibited two distinct peaks (Fig. 3A), suggesting that all GDP samples are crude polysaccharide mixtures containing both high- and low-molecular-weight components. A standard curve was established using dextran standards (Mw: 4.3–275 kDa) with the equation *y* = −0.3629*x* + 10.9129 (*R*^2^ = 0.9952). Based on this curve, the molecular weights (Mw) of the low-molecular-weight fractions in GDP-W, GDP-A, and GDP-E were determined to be 27.78 kDa, 19.98 kDa, and 5.744 kDa, respectively. According to approximate peak area comparisons, GDP-W was rich in low-molecular-weight polysaccharides, whereas GDP-A contained a larger proportion of high-molecular-weight polysaccharides.

The monosaccharide composition was analyzed based on the standard curves of seven monosaccharide standards and the chromatographic results of the mixed standard. The chromatograms and quantitative results of the GDPs are presented in Fig. 3B and Table 1, respectively. GDP-W and GDP-E exhibited similar monosaccharide profiles, both primarily composed of Ara and GalA, though in differing ratios. The molar ratios of Ara to GalA were 41.40: 42.18 in GDP-W and 51.64: 29.72 in GDP-E. In contrast, GDP-A displayed a distinct composition, dominated by Ara, Man, and Glc, with molar ratios of 23.19: 44.48:12.17, respectively. Notably, the proportion of Man in GDP-A (44.48%) was strikingly higher than that in GDP-W (0.92%) and GDP-E (0.99%).

FT-IR spectroscopy was used for the preliminary structural characterization of polysaccharides. It requires only a small amount of sample and provides valuable information about functional groups and glycosidic linkages. As shown in Fig. 3D, all three polysaccharides showed characteristic carbohydrate absorption bands: a strong broad peak around 3323 cm^−1^, attributed to O–H stretching vibrations, and a weak peak near 2928 cm^−1^, attributed to C–H stretching vibrations [27]. Both GDP-W and GDP-E exhibited absorption peaks near 1735 cm^−1^ and 1605 cm^−1^, which are indicative of esterified and non-esterified carbonyl (C=O) stretching vibrations, respectively. GDP-A showed only a strong peak at 1605 cm^−1^, implying a difference in the degree of esterification, likely due to the extraction method [28]. Additional peaks observed at 1417, 1323, 1228, and 1039 cm^−1^ were assigned to C–O stretching vibrations. The presence of a peak at 896 cm^−1^ suggests β-glycosidic linkages [29], indicating that GDP-A mainly contains β-type glycosidic bonds.

**Fig. 4:**
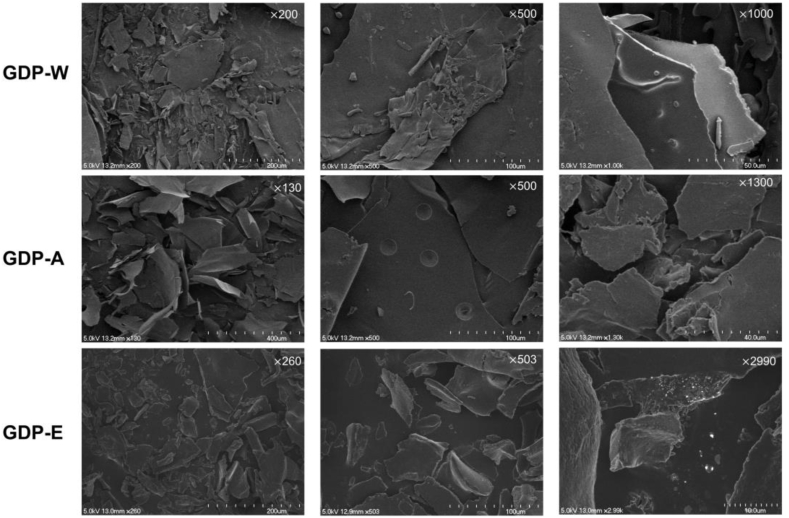
SEM characterization of GDPs.

SEM images (Fig. 4) showed that both GDP-A and GDP-E have rough, fragmented, and irregular morphological features, with GDP-A exhibiting the most compact and sheet-like structure. The alkaline method causes greater disruption to plant cell walls, leading to pronounced differences in the size and morphology of the extracted polysaccharides.

These results indicated that hot water extraction primarily releases free, water-soluble polysaccharides with low molecular weight, whereas alkaline extraction disrupts cell wall linkages, liberating larger and more tightly bound structural polysaccharides. Monosaccharide composition analysis suggested that the polysaccharides extracted from *G. dahurica* are primarily pectic in nature, mainly composed of Ara and GalA. This finding is consistent with previous studies. For instance, Ji et al. also obtained two polysaccharides (GDP-1 and GDP-2) from *G. dahurica* via hot water extraction and subsequent ion-exchange chromatography, which were predominantly constituted of Ara and GalA. GDP-1 contained 53.5% Ara and 30.8% GalA, while GDP-2 comprised 33.9% Ara and 48.5% GalA [15]. Minor variations in monosaccharide profiles have been reported among different *Gentiana* species. Cheng et al. identified the presence of not only Ara and GalA but also considerable amounts of Glc and Gal in polysaccharides from *G.* scabra bge [30]. Similarly, Zou et al. reported a high Gal content in polysaccharides from *G. crassicaulis*, alongside Ara and GalA [31]. Despite these slight differences, it can be concluded that Ara and GalA represent the characteristic monosaccharide components of polysaccharides within the *Gentiana* genus.

The differential extraction methods caused varying degrees of damage to the plant cell wall. Hot water mainly solubilizes pectic polysaccharides from the primary cell wall, while strong alkaline conditions cleave hydrogen and ester bonds, thus releasing structural polysaccharides such as those rich in Man and Glc from the secondary cell wall. The markedly increased mannose content in GDP-A suggests that alkali extraction may favor the release of certain mannose-rich polysaccharide fractions in *Gentiana dahurica*. Previous studies have shown that alkali extraction of polysaccharides from different plant roots does not necessarily lead to an increase in mannose content [32,33]. Therefore, the high-mannose feature observed in the present study should not be simply regarded as a general consequence of alkali extraction itself, but is more likely the result of the combined effects of the intrinsic polysaccharide composition of *Gentiana dahurica* and the selectivity of alkali extraction.

### 3.3 *Invitro* bioactivity evaluation of GDPs

#### 3.3.1 Antioxidant activity of GDPs

The excessive production of ROS can induce oxidative stress, disrupt the antioxidant defense system, and contribute to inflammation, aging, metabolic disorders, and cancer [34]. Consequently, there is significant interest in developing effective, safe, and natural antioxidants. Previous work by Ji et al. showed that the polysaccharides GDP-1 and GDP-2 from *G. dahurica* possess strong free radical scavenging abilities [15]. Building on this, the present study evaluates how different extraction methods affect the antioxidant activities of *G. dahurica* polysaccharides in vitro.

**Fig. 5:**
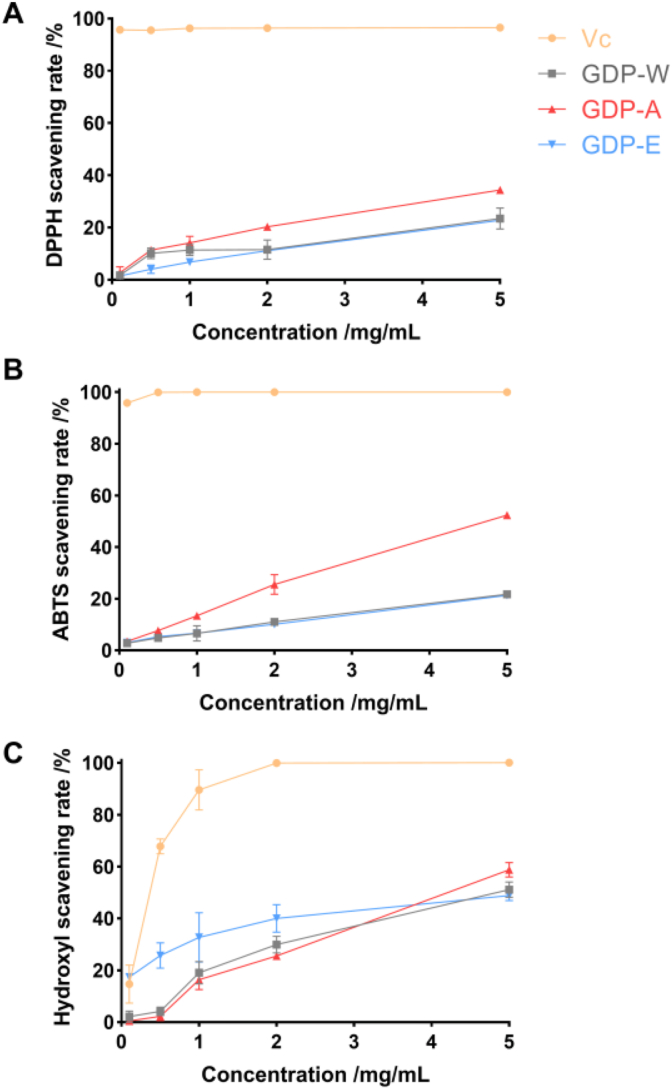
Scavenging activities of GDPs against DPPH (A), ABTS (B), and Hydroxyl (C) radicals.

The free radical scavenging rates of GDPs increased with concentration (Fig. 5). The GDP-A showed significantly greater ability to scavenging DPPH and ABTS over a large concentration range. GDP-A has comparable hydroxyl radical scavenging ability to GDP-W which was lower than GDP-E at concentration of 0.1 - 2 mg/mL but greater than GDP-E at 5 mg/mL. The ABTS radical scavenging rates of GDP-W and GDP-E were below 50%, while that of GDP-A reached 52.43%. At the same concentration, the DPPH radical scavenging rates of all GDPs were lower than 50%, and the hydroxyl radical scavenging rates were approximately 50%. Overall, all GDPs exhibited concentration-dependent radical scavenging activity. Among them, GDP-A exhibited the strongest antioxidant capacity, highlighting the impact of extraction methods on biological activity. The differences in antioxidant activity among GDPs may be attributed to in their distinct chemical structures. The enhanced activity of GDP-A is likely related to its high Man content (44.48%) [35].

#### 3.3.2 The effects of GDPs on HaCaT cells exposed to UVB radiation

**Fig 6:**
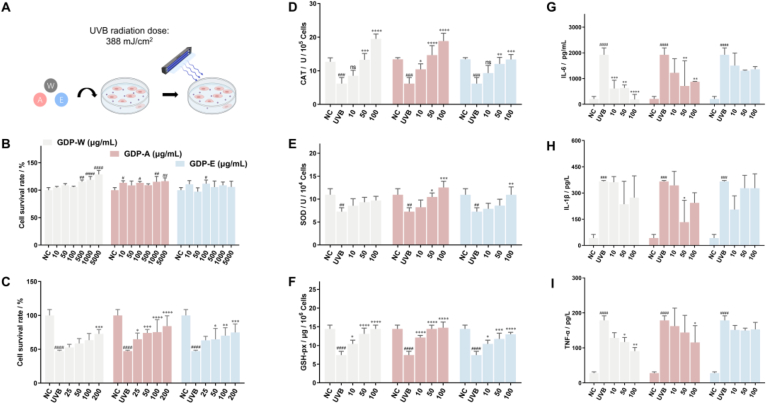
Effects of GDPs on HaCaT cells exposed to UVB radiation A. Schematic of the UVB-induced cell model; B. Cytotoxicity of GDPs; C. Cell viability; (D-F) Oxidative stress markers: CAT, SOD, and GSH-px activities; (G-I) Pro-inflammatory cytokines: IL-6, IL-1β, and TNF-α levels. Note: ^#^*p* < 0.05 vs Normal Control; ∗*p* < 0.05 vs UVB Model.

Before conducting in vivo experiments, cytotoxicity assessment of natural polysaccharides should be conducted. GDPs showed no cytotoxicity toward HaCaT cells within a certain concentration range (Fig. 6B). The protective effect of GDPs against UVB-irradiation was further evaluated (Fig. 6C). The results indicated that UVB irradiation significantly reduced cell survival. Pretreatment with GDP-W, GDP-A, and GDP-E increased cell viability. Notably, significant increases in HaCaT cell survival were observed at concentrations of 200 μg/mL for GDP-W, 25 μg/mL for GDP-A, and 50 μg/mL for GDP-E.

Moreover, the levels of CAT, SOD, and GSH-Px were measured to evaluate antioxidant capacity (Fig. 6D, 6E, and 6F). UVB irradiation significantly decreased the levels of these antioxidant enzymes. All three biomarkers showed concentration-dependent increases after pre-incubation with GDPs. Notably, the method of extraction significantly influenced the antioxidant potency of the polysaccharides. GDP-A and GDP-W exhibited comparable effect in increasing the levels of CAT and GSH-px, which was significantly greater than that effect of GDP-E. GDP-A showed a significantly greater effect on increasing the level of SOD than GDP-W and GDP-E. This is consistent with the in vitro radical scavenging results. The levels of inflammatory factors were measured too (Fig. 6G, 6H, and 6I). GDP-W showed significantly greater efficacy in decreasing the production of IL-6 and TNF-α than that of GDP-A and GDP-E. GDP-A showed greater effect on decreasing the levels of IL-1β than that of GDP-W and GDP-E.

**Fig. 7:**
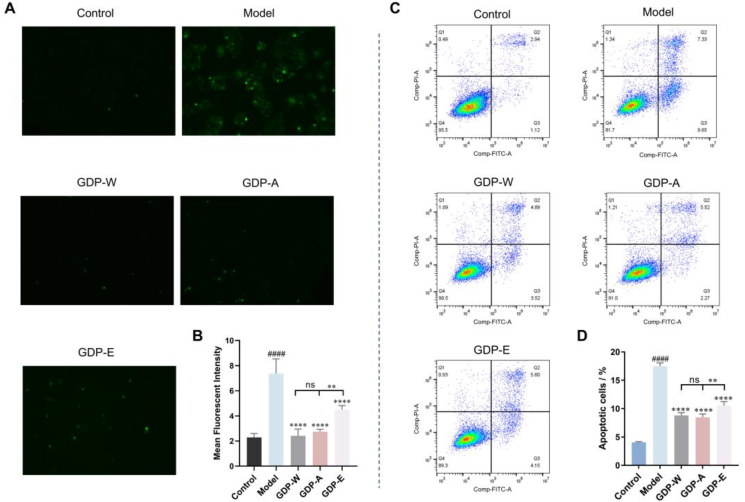
Effects of GDPs on intracellular ROS levels and apoptosis. A. Results of intracellular ROS; B. Statistical analysis of ROS; C. Results of apoptosis; D. Quantification of apoptosis rate. Note: ^#^*p* < 0.05 vs Control; ∗*p* < 0.05 vs Model.

Furthermore, the levels of ROS were used to investigate the antioxidant activity of GDPs in vitro. Intracellular ROS levels were increased significantly after UVB irradiation compared to the control group (Fig. 7A). Pretreatment with 200 μg/mL GDPs before UVB exposure markedly reduced intracellular ROS levels. These results indicated that GDPs provide protection against UVB-induced damage. GDP-A and GDP-W showed greater efficacy than GDP-E.

#### 3.3.3 Effects of GDPs on apoptosis

Apoptosis plays a critical role in cell growth. ROS-induced oxidative damage can trigger the DNA damage, activate downstream signaling pathways that inducing the execution of the apoptotic program. Consistent with this mechanism, UVB irradiation significantly increased the apoptotic cell ratio from 4.06 % in the control group to 16.98 % (Fig. 7C). Pretreatment with GDPs markedly attenuated UVB-induced apoptosis, reducing the apoptotic rate to 8.41% in the GDP-W group, 7.79% in the GDP-A group, and 9.75% in the GDP-E group. These results support the conclusion that GDPs inhibit UVB-induced apoptosis by reducing ROS levels and regulating immune reactions. Overall, GDPs exhibited both preventive and therapeutic effects in the HaCaT cell model of UVB-induced damage, with GDP-A showing the most prominent biological activity.

### 3.4 Effect of GDPs on zebrafish embryos exposed to UVA

**Fig 8:**
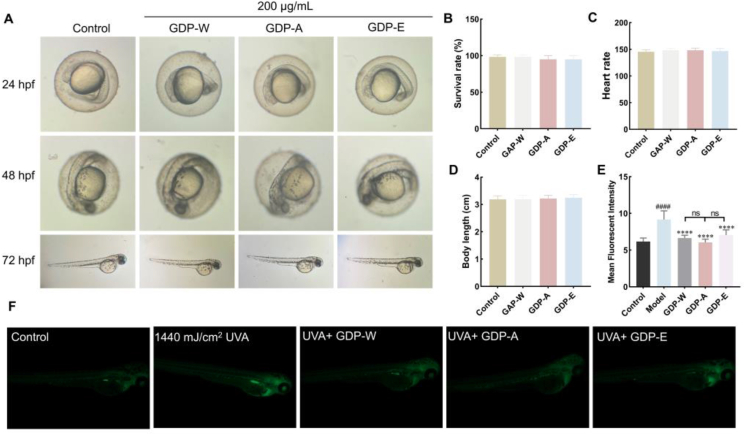
The anti-photoaging effects of GDPs in UVA-exposed zebrafish. A: Images of embryonic development; B-D: Larvae after 72 h GDPs exposure: survival rate (B), heart rate (C), and body length (D); E. Quantitative analysis of ROS levels; F. Representative fluorescence images of ROS. Note: ^#^*p* < 0.05 vs Control; ∗*p* < 0.05 vs Model.

Zebrafish, serving as a model organism, are an ideal in vivo model for studying the biological effects of ultraviolet radiation because of their transparent embryos, which allow direct observation of UV effects, rapid development, and high genetic homology with humans [36]. Prior to the UV damage experiments, the impact of GDPs exposure on acute toxicity parameters was evaluated. The results showed no significant difference in the survival rates of embryos exposed to GDPs compared to those in the control group (Fig. 8B). After 72 h of culture, the heart rate (Fig. 8C) and body length (Fig. 8D) of the larval were unaffected. The effects of GDPs on zebrafish were further investigated by examining embryonic development at 24, 48, and 72 hpf (Fig. 8A). Body morphology and pigmentation patterns served as critical markers for developmental assessment. No treatment-related malformations were observed in GDP-treated embryos, which maintained normal development characterized by an intact body axis, yolk sac, and appropriate pigmentation. GDPs demonstrated high safety in zebrafish embryos.

UVA, a long-wave ultraviolet radiation with a strong penetrating ability, is often associated with skin photoaging following long-term exposure. To systematically evaluate the protective effects of GDPs against UV radiation, a zebrafish model of UVA radiation damage was used to assess the oxidative stress in vivo. UVA irradiation significantly enhanced green fluorescence in the zebrafish, indicating a significant increase in ROS production (Fig. 8F). Treatment with 200 μg/mL GDPs significantly reduced fluorescence intensity. The three polysaccharides showed similar effects, with no significant differences among them (Fig. 8E). These results demonstrate that treatment with a certain concentration of GDPs reduces UVA-induced ROS production.

### 3.5 *Invivo* therapeutic evaluation of GDP-A on UVB-induced skin damage

**Fig. 9:**
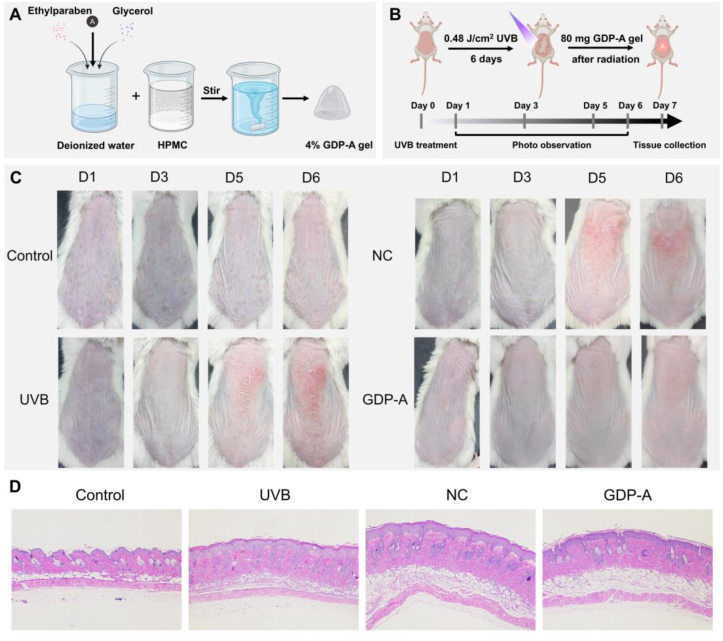
Skin appearance and tissue sections of mice in each group after UVB irradiation. A: Schematic diagram of gel preparation; B: Establishment and treatment of mouse photoaging model; C: Skin appearance photos; D: H&E-stained skin tissue sections.

GDP-A demonstrated relatively superior protective effects in both cellular and zebrafish UV radiation damage. To further assess the therapeutic potential of GDP-A in vivo, a mouse model of photoaging induced by UVB was developed. Application of polysaccharide solutions directly onto the damaged dorsal skin of mice often leads to poor adherence and incomplete absorption. In contrast, gel offer higher viscosity, prolonging the retention of polysaccharides on the skin and enhancing transdermal absorption, thereby ensuring the effective delivery [37]. Therefore, a gel formulation of GDP-A was developed for this study. Mice dorsal skin observations on days 1, 3, 5, and 6 revealed that UVB irradiation induced a range of skin reactions, including redness, scaling, wrinkling, roughness, and dullness (Fig. 9C). After six consecutive days of treatment, the NC (blank gel control) group still exhibited significant symptoms such as erythema, deepened wrinkles, and coarse skin texture. In contrast, the GDP-A group showed noticeably smoother skin, normal coloration, improved elasticity, and overall better recovery.

H&E-stained sections of dorsal mice skin revealed well-organized skin texture and tightly arranged collagen fibers in the control group (Fig. 9D). In contrast, the UVB group exhibited significant epidermal and spinous layer thickening, pale staining in the superficial dermis, fragmented collagen fibers, nuclear rupture, and inflammatory cell infiltration. The blank gel group showed no notable improvement compared to the UVB model. By comparison, the GDP-A treated group demonstrated significantly reduced epidermal and spinous layer thickness, relatively orderly collagen fiber arrangement, and restoration of epidermal thickness to near-normal levels.

**Fig. 10:**
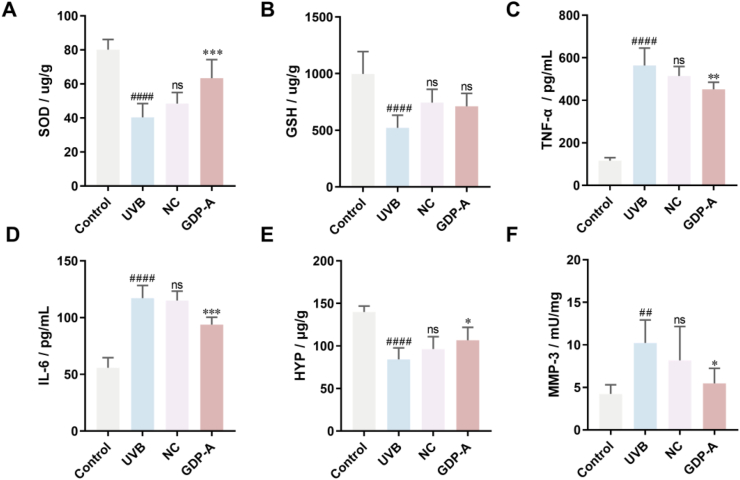
Therapeutic effects of GDP-A on UVB-induced skin damage. A. SOD; B. GSH; C. TNF-α; D. IL-6; E. HYP; F. MMP-3; Note: ^#^*p* < 0.05 vs Control; ∗*p* < 0.05 vs UVB.

Moreover, UV radiation often induces oxidative stress, and the level of skin oxidative damage is commonly used as an evaluation indicator for UV radiation therapy [38]. The metalloenzyme SOD, which is ubiquitous in living organisms, is central to redox balance [39]. GSH is a natural antioxidant product capable of scavenging free radicals in the body [40]. Compared with the control group, UVB irradiation significantly reduced the levels of both SOD and GSH (Fig. 10A and B). The blank gel group showed no significant improvement compared to the UVB model. Treatment with the GDP-A gel significantly increased SOD activity, while no significant change was observed in GSH levels.

Long-term UV irradiation can lead to local skin damage and even reduce the immune response capacity of the systemic immune system. The changes of IL-6 and TNF-α are closely related to this process [41]. After 6 days of treatment, the contents of TNF-α and IL-6 showed that the TNF-α content in skin tissues of the UVB group and the blank gel group significantly increased to 563.7 pg/mL and 514.40 pg/mL, respectively (Fig. 10C and D). In contrast, the GDP-A treatment group exhibited the most significant decrease in TNF-α, with a level of 451.63 pg/mL. A similar trend was observed for IL-6 levels across the groups.

Inhibition of collagen synthesis is a feature of photoaging. Hydroxyproline (HYP), a characteristic amino acid in collagen, is commonly used as an indicator to evaluate collagen content in the skin [42]. UVB irradiation induces collagen depletion in the skin, which over time contributes to increased wrinkle formation. The HYP content in mouse skin tissues was significantly decreased in the UVB group (Fig. 10E). GDP-A treatment significantly increased HYP levels.

Further, the mechanism of action was explored via measuring the expression of MMP-3. UVB radiation activates the MAPK/AP-1 and NF-κB signaling pathways, and MMP-3 is a well-established downstream target that can be upregulated by these pathways [43]. It has been reported that MMP-3 can degrade skin elastin and promote the breakdown of collagen and elastic fibers [44]. The level of MMP-3 was significantly increased (10.22 mU/mg) in the UVB model group. GDP-A treatment effectively suppressed its expression (Fig. 10F). These results demonstrated that *G. dahurica* polysaccharides can effectively suppress UV-induced overexpression of MMP-3, thereby helping to maintain the structure of skin collagen and alleviate photodamage.

GDP-A showed strong antioxidant activity and photoprotective effects. Its relatively high mannose content suggests that mannose-rich structures may contribute to its bioactivity. Some polysaccharides in plant cell walls exist in tightly bound forms, alkali treatment may disrupt hydrogen bond and ester bond interactions between polysaccharides and the cell wall matrix [45]. This process may promote the release of bound polysaccharides. In addition, alkali-induced de-esterification can increase the negative charge of carboxyl groups and alter molecular weight and chain conformation [46,47]. These changes can further affect polysaccharide solubility. They may also make some bioactivity-related side chains or charged domains more accessible. The bioactivity of polysaccharides is closely associated with their molecular weight, conformation, and solubility. Therefore, structural differences among polysaccharides obtained by different extraction methods may further contribute to differences in their antioxidant and photoprotective activities.

## 4 Limitation

This study has several limitations. The fine structures of GDP-W, GDP-A, and GDP-E were not further elucidated, particularly by NMR and linkage analysis, which are important for clarifying glycosidic linkages, residue sequence, and chain conformation of polysaccharides. Consequently, the structure–activity relationship could not be fully validated. Although GDP-A showed superior antioxidant and photoprotective activities, the present results only indicate a preliminary association between its structural characteristics and bioactivity. This association has not been rigorously validated. Since extraction methods can alter polysaccharide structure and thereby influence bioactivity, further in-depth structural characterization and targeted validation experiments are still required.

## 5 Conclusion

This study employed three extraction methods to isolate polysaccharides from *G. dahurica*, obtaining water-extracted polysaccharide (GDP-W) and enzyme-extracted polysaccharide (GDP-E), both primarily composed of Ara and GalA, as well as alkaline-extracted polysaccharide (GDP-A) dominated by Man. GDPs exhibited significant antioxidant activity and alleviated the UVB-induced cell damage. GDPs significantly reduced the UVA-induced increase in ROS levels in zebrafish. In a UVB-induced photoaging mouse model, GDP-A alleviated oxidative stress and inflammation, reduced collagen degradation, and suppressed MMP-3 expression. Thus, the alkaline extracted polysaccharides (GDP-A) had a unique chemical composition and effectively alleviated skin damage. These findings further elucidate the extraction method-structure-activity relationship of *G. dahurica* polysaccharides and provide valuable references for their systematic development and application in the pharmaceutical and cosmetic industries.

## CRediT authorship contribution statement

**Jiahui Xia:** Writing – original draft, Methodology, Investigation, Data curation. **Muhammad Aamir Zohaib:** Writing – original draft, Methodology, Investigation, Data curation. **Xiaopeng Su:** Writing – original draft, Methodology, Investigation, Data curation. **Wen Ji:** Writing – original draft, Methodology, Investigation, Data curation. **Tengfei Hu:** Investigation. **Jianqin Zhou:** Project administration. **Sheng Tian:** Writing – review & editing, Conceptualization. **Duxin Li:** Writing – review & editing, Supervision, Conceptualization.

## Ethics approval

The mice used in this study were purchased from GemPharmatech Co., Ltd. (certificate number: A202401180152). All animal experiments were approved by the Animal Ethics Committee of Soochow University (approval number: 202401A0119).

## Declaration of generative AI in scientific writing

Not applicable.

## Funding information

This work was supported by the 10.13039/501100012246Priority Academic Program Development of Jiangsu Higher Education Institutions (10.13039/501100012246PAPD).

## Declaration of competing interest

The authors declare that they have no known competing financial interests or personal relationships that could have appeared to influence the work reported in this paper.

## Data Availability

The data that support the findings of this study are available from the corresponding author upon reasonable request.
